# Bivalent metal ions induce formation of α-synuclein fibril polymorphs with different cytotoxicities

**DOI:** 10.1038/s41598-022-15472-4

**Published:** 2022-07-13

**Authors:** Deyhim Atarod, Fatemeh Mamashli, Atiyeh Ghasemi, Faezeh Moosavi-Movahedi, Mitra Pirhaghi, Hadi Nedaei, Vladimir Muronetz, Thomas Haertlé, Jörg Tatzelt, Gholamhossein Riazi, Ali Akbar Saboury

**Affiliations:** 1 Institute of Biochemistry and Biophysics, University of Tehran, Tehran, Iran; 2 Belozersky Institute of Physico-Chemical Biology, Lomonosov Moscow State University, Moscow, Russia 119991; 3 National Institute of Agronomic and Environmental Research, 44316 Nantes, France; 4 Department Biochemistry of Neurodegenerative Diseases, Institute of Biochemistry and Pathobiochemistry, Ruhr University Bochum, Bochum, Germany; 5 Cluster of Excellence RESOLV, Ruhr University Bochum, Bochum, Germany

**Keywords:** Biochemistry, Biophysics, Neuroscience

## Abstract

α-Synuclein (α-Syn) aggregates are key components of intracellular inclusion bodies characteristic of Parkinson’s disease (PD) and other synucleinopathies. Metal ions have been considered as the important etiological factors in PD since their interactions with α-Syn alter the kinetics of fibrillation. In the present study, we have systematically explored the effects of Zn^2+^, Cu^2+^, Ca^2+^, and Mg^2+^ cations on α-Syn fibril formation. Specifically, we determined fibrillation kinetics, size, morphology, and secondary structure of the fibrils and their cytotoxic activity. While all cations accelerate fibrillation, we observed distinct effects of the different ions. For example, Zn^2+^ induced fibrillation by lower *t*_lag_ and higher *k*_app_ and formation of shorter fibrils, while Ca^2+^ ions lead to formation of longer fibrils, as evidenced by dynamic light scattering and atomic force microscopy studies. Additionally, the morphology of formed fibrils was different. Circular dichroism and attenuated total reflection-Fourier transform infrared spectroscopies revealed higher contents of β-sheets in fibrils. Interestingly, cell viability studies indicated nontoxicity of α-Syn fibrils formed in the presence of Zn^2+^ ions, while the fibrils formed in the presence of Cu^2+^, Ca^2+^, and Mg^2+^ were cytotoxic. Our results revealed that α-Syn fibrils formed in the presence of different divalent cations have distinct structural and cytotoxic features.

## Introduction

α-Synuclein (α-Syn) is a highly conserved 140-residue intrinsically disordered protein, which is abundantly present in presynaptic terminals in the brain^1^. Misfolding and aggregation of α-Syn in neurons and glial cells, as intracellular inclusions called Lewy bodies, is a pathological characteristic of a group of neurodegenerative diseases called synucleinopathies, mainly among them is Parkinson’s disease (PD)^2,3^. Others include Lewy body dementia (LBD)^4^ and multiple system atrophy (MSA)^5^. α-Syn is composed of three separate regions: 1) the amphipathic N-terminus (residues 1–60), which is positively charged and adopts an α-helical structure when interacting with biological membranes; 2) the central highly hydrophobic self-aggregating sequence called NAC (non-amyloid β component; residues 61–95), which is the part involved in the formation of β-sheet during fibrillation; and 3) the acidic C-terminus (residues 96–140), which contains a number of negatively charged residues and is the main binding site for metal ions^6^. α-Syn is natively unfolded and lacks a unique structure and adopts multitude of conformations with no persistent secondary structure in its native monomeric state^7,8^. Although still controversial, it is believed that α-Syn plays a role in regulation of synaptic vesicle recycling via interacting with negatively charged lipids, where metal ions can interfere^9^. It has been shown that Ca^2+^ binding by α-Syn can change the charge distribution of the negatively charged C-terminus and consequently modulate the α-Syn-membrane interactions^9^.

On the other hand, interactions between α-Syn and metal ions have been indicated to favor α-Syn folding, oligomerization, aggregation, and fibrillation^10,11^. High concentrations of Zn^2+^, Cu^2+^, and Ca^2+^ were detected in α-Syn fibrillar deposits in parkinsonian *substantia nigra*^12,13^. Therefore, binding of various metal ions to α-Syn has been largely explored in order to elucidate the molecular mechanism underlying α-Syn fibrillation acceleration and pathogenesis of induced diseases^10,12,14,15^. Mapping the α-Syn-Zn^2+^ interactions using NMR spectroscopy located the ions binding site around Asp121, Asn122 and Glu123 with Asp121 as the main anchoring site and another lower affinity binding site on His50 in the unstructured N terminal domain^16^. Studies of the binding sites of Cu^2+^ ions on α-Syn revealed that the N terminus is the primary binding site involving His50 as the main anchor, while C terminus with negatively charged residues acts as a secondary binding site with a much lower binding affinity^6^. Ca^2+^ and Mg^2+^ were found to bind the acidic C terminal domain^17–19^.

In spite of the efforts for identifying the binding sites of metal ions on α-Syn and their accelerating effect on α-Syn fibrillation, a comparison of the cytotoxicities of the fibrils induced by metal ions has been remained largely unexplored. Therefore, in the current comparative study we scrutinized more precisely the morphology, structures, and cytotoxicity of α-Syn fibrils formed in the presence of Zn^2+^, Cu^2+^, Ca^2+^, or Mg^2+^ ions. We tried to answer this question that whether polymorphically different α-Syn fibrils formed in the presence of various metal ion inducers could have different toxicities. The findings of this research can pave the way for the elucidation of the role of the diverse inducers in the α-Syn fibrillation and PD pathology.

## Results

### Metal ions accelerated α-synuclein fibril formation

**Figure 1:**
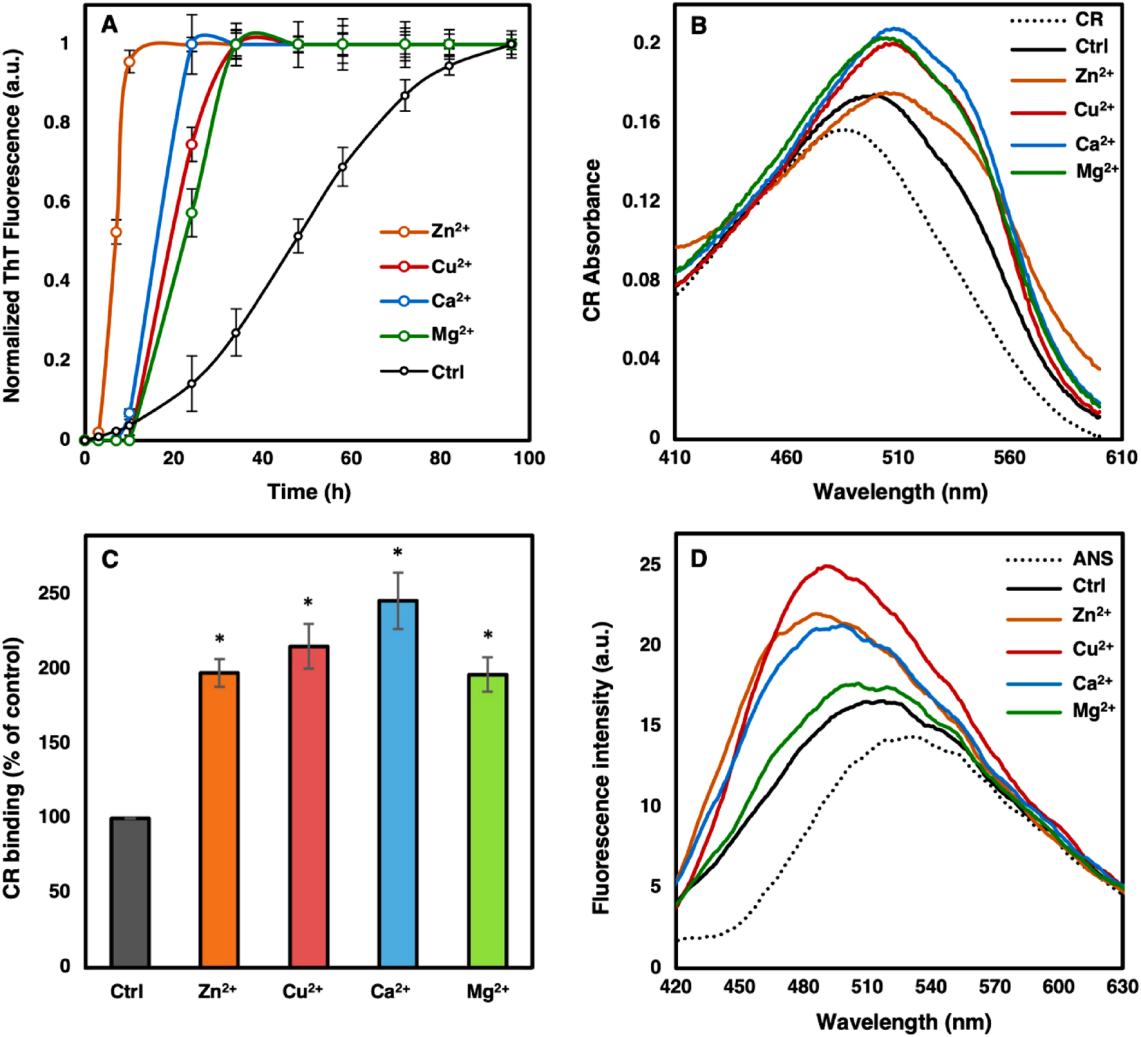
α-Syn fibril formation in the presence and absence of each cation. 100 µM α-Syn was incubated under constant shaking (1000 rpm) in 20 mM Tris buffer (pH 7.5) supplemented with 500 µM of the mentioned metal ions at 37 °C. As a control (Ctrl), α-Syn was incubated in Tris buffer alone. (**A**) For kinetic studies, fibril formation was monitored by 25 µM ThT fluorescence excited at 450 nm. (**B**) Congo Red binding absorbance spectra of α-Syn fibrils formed in the presence and absence of the metal ions. (**C**) The amount of fibrils formed compared to control calculated based on bound CR using Eq. (2). (**D**) ANS fluorescence measurements were performed on end stage fibrils of each sample. Error bars present the standard deviation of three independent experiments. CR: Congo red solution. ANS: ANS solution.

Effect of metal ions on kinetic of α-Syn amyloid formation was evaluated using the standard ThT fluorescence assay (Fig. 1A). ThT data were normalized and then fitted using Eq. (1) (Table 1). According to the obtained results, presence of Zn^2+^, Cu^2+^, Ca^2+^, and Mg^2+^ accelerated α-Syn fibril formation. Presence of metal ions led to a significant decrease in *t*_lag_ and increase in *k*_app_ of α-Syn fibrillation compared to the control condition in tris buffer (*p* < 0.05). A considerable decrease in the lag time of aggregation (*t*_lag_) was observed for the α-Syn fibrillation kinetics in presence of Zn^2+^ ions, which was accompanied by a higher *k*_app_ compared to the control and other metal ions (*p* < 0.05). Fibril formation in the presence of Cu^2+^, Ca^2+^, and Mg^2+^ was nearly parallel and followed a similar trend with the Mg^2+^ sample having a slower rate compared to the other two cations (*p* < 0.05). Control sample in the absence of the ions has a lag phase of 18 h. Interestingly, Zn^2+^ seemed to enhance the kinetic of α-Syn fibril formation by nearly 5 times compared to the control sample. Statistical analysis of the calculated *t*_lag_ and *k*_app_ showed significant differences among the kinetic parameters of fibrillation between various metal ions.

**Table 1:** Kinetic parameters of α-Syn fibril formation in presence and absence of each metal ion. The parameters were calculated based on kinetic studies displayed in Fig. 1 using Eq. (1). * *p* < 0.05 significant difference compared to the control group. **p < 0.05 significant difference compared to the Zn^2+^ group. ***p < 0.05 significant difference compared to the Cu^2+^ group. ****p < 0.05 significant difference compared to the Ca^2+^ group.

	Control	Zn^2+^	Cu^2+^	Ca^2+^	Mg^2+^
t_lag_ (h)	18 ± 0.26	2 ± 0.56	9 ± 0.20	6 ± 0.26	13.5 ± 0.50
Statistical significance vs. control		*	*	*	*
Statistical significance vs. Zn^2+^			**	**	**
Statistical significance vs. Cu^2+^				***	***
Statistical significance vs. Ca^2+^					****
*k*_app_ (h^-1^)	0.07 ± 0.01	0.33 ± 0.05	0.12 ± 0.06	0.25 ± 0.05	0.17 ± 0.03
Statistical significance vs. control		*	*	*	*
Statistical significance vs. Zn^2+^			**	**	**
Statistical significance vs. Cu^2+^				***	***
Statistical significance vs. Ca^2+^					****

Then the Congo red (CR) binding assay was performed to further confirm α-Syn fibril formation and to quantitatively evaluate the formation of the β-sheets during fibrillation. As shown in Fig. 1A, presence of amyloid fibrils in the control sample caused a higher CR absorbance accompanied by a red shift. Interestingly, we observed a shoulder at around 540 nm for Zn^2+^, Cu^2+^, Ca^2+^, and Mg^2+^ samples, what can be assigned to the higher presence of amyloid fibrils. Molar concentration of the bound CR, as a quantitative criterion for the amount of amyloid fibrils, was calculated using Eq. (2) and presented in Fig. 1C. According to the obtained results, presence of Zn^2+^, Cu^2+^, Ca^2+^, and Mg^2+^ led to a significant increase in the amount of amyloid fibrils compared with the control sample. The presence of metal ions led to higher yield of α-Syn fibril formation. However, no significant difference was detected between CR binding of fibrils formed in the presence of various metal ions (*p* > 0.05).

Fibrils of α-Syn formed in the presence or absence of Zn^2+^, Cu^2+^, Ca^2+^, and Mg^2+^ were also analyzed using ANS binding assay^20^. Based on the results displayed in Fig. 1D, fibrils in the control sample resulted in a minor increase in ANS fluorescence intensity along with a blue shift indicative of more hydrophobic clusters formed upon fibrillation. Presence of Mg^2+^ led to similar changes. However, Zn^2+^, Cu^2+^, and Ca^2+^ resulted in greater changes in hydrophobic patches of α-Syn fibrils.

**Figure 2:**
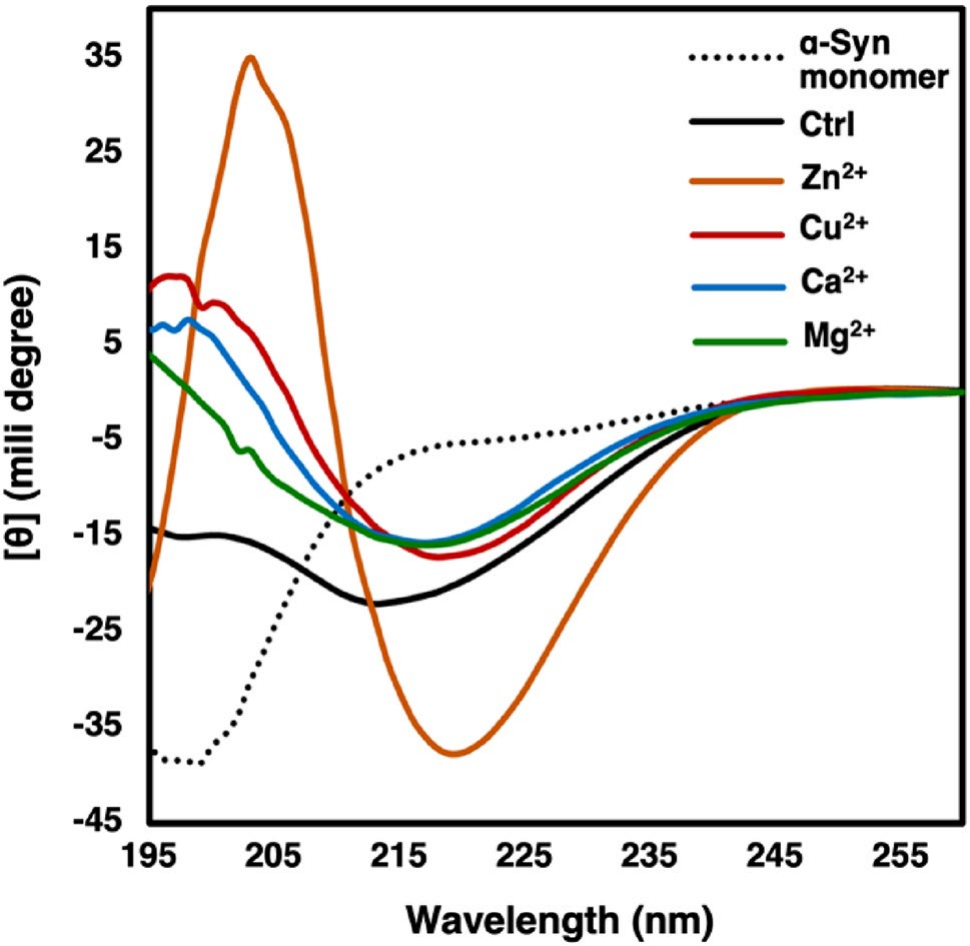
Far-UV CD spectra of α-Syn monomer and fibrils formed in the presence and absence of each cation. The fibrils formed by 100 µM α-Syn in 20 mM Tris buffer (pH 7.5) or in presence of 500 µM of the metal cations were analyzed. The measurements were carried out in room temperature (25 °C). See materials and methods for details.

In order to confirm the presence of β-sheet structure in the aggregates, far-UV CD and attenuated total reflection-Fourier transform infrared (ATR-FTIR) spectroscopies were performed. According to the CD spectra presented in Fig. 2, fibrils formed in the presence of cations showed a clear change from random-coil structure, as characterized by the negative ellipticity at wavelength below 200 nm, to β-sheet form, as characterized by the negative ellipticity at 218–220 nm along with the positive ellipticity at wavelength 195 nm^21^. Fibrils formed in tris buffer, in the absence of the metal ions, display the negative ellipticity at 218–220 nm along with a higher ellipticity, compared to α-Syn monomer, below 200 nm. Our results on CD spectroscopy reveals formation of β-sheets in all the fibril samples.

**Figure 3:**
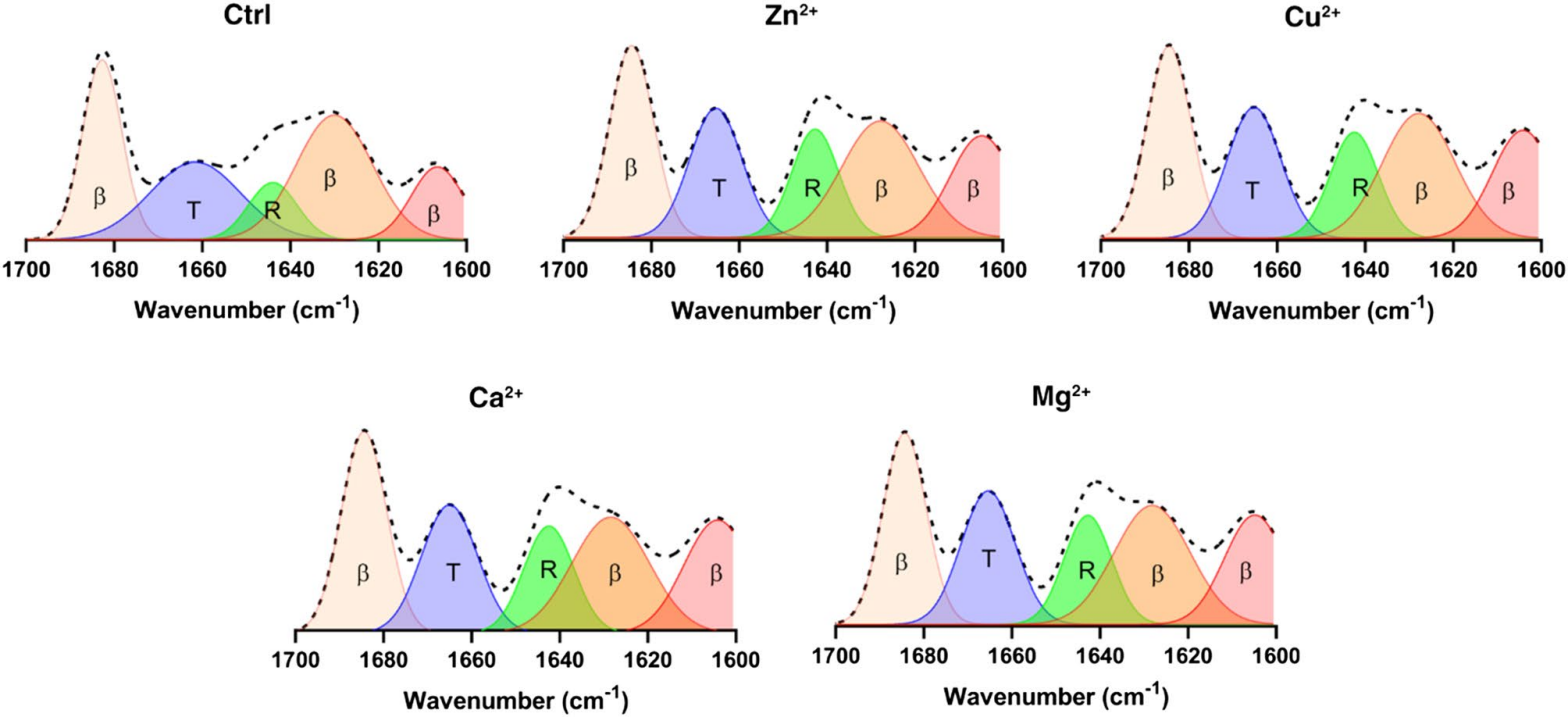
ATR-FTIR spectra and their deconvolution of α-Syn fibrils formed in the presence and absence of each cation. Amide I region (1700–1600 cm^−1^) of each samples’ spectrum are displayed. In each panel the un-deconvoluted spectrum appears as a dashed line, while the deconvoluted spectrum are shown as colorful graphs. The water-subtracted data were base-line corrected, smoothed, and normalized using GraphPad Prism 8. The amide I region (1700–1600 cm^−1^) were fitted based on a Gaussian model using OriginPro 2022. The colorful images were prepared for presentation in the manuscript using GraphPad Prism 8. β: β-sheet; R: random coil; T: β-turn.

The ATR-FTIR spectra recorded for α-Syn fibrils formed in the presence and absence of various cations and their deconvolution displayed in Fig. 3 indicate the distinctive bands at $\sim$ 1606, $\sim$ 1629, and $\sim$ 1684 cm^−1^ in the amide I region of all the fibril samples revealing the dominance of β sheets (at least 61%), which is in accordance with previous studies^22–25^. Furthermore, a band at $\sim$ 1660 cm^−1^ was detected in all the fibrils samples, which is attributed to the presence of β turns^23^. According to the deconvolution results, contents of β-sheets in the fibrils formed in the presence of cations were slightly higher compared to the fibrils formed in Tris buffer.

### Various metal ions induced formation of different α-synuclein fibril polymorphs

**Figure 4:**
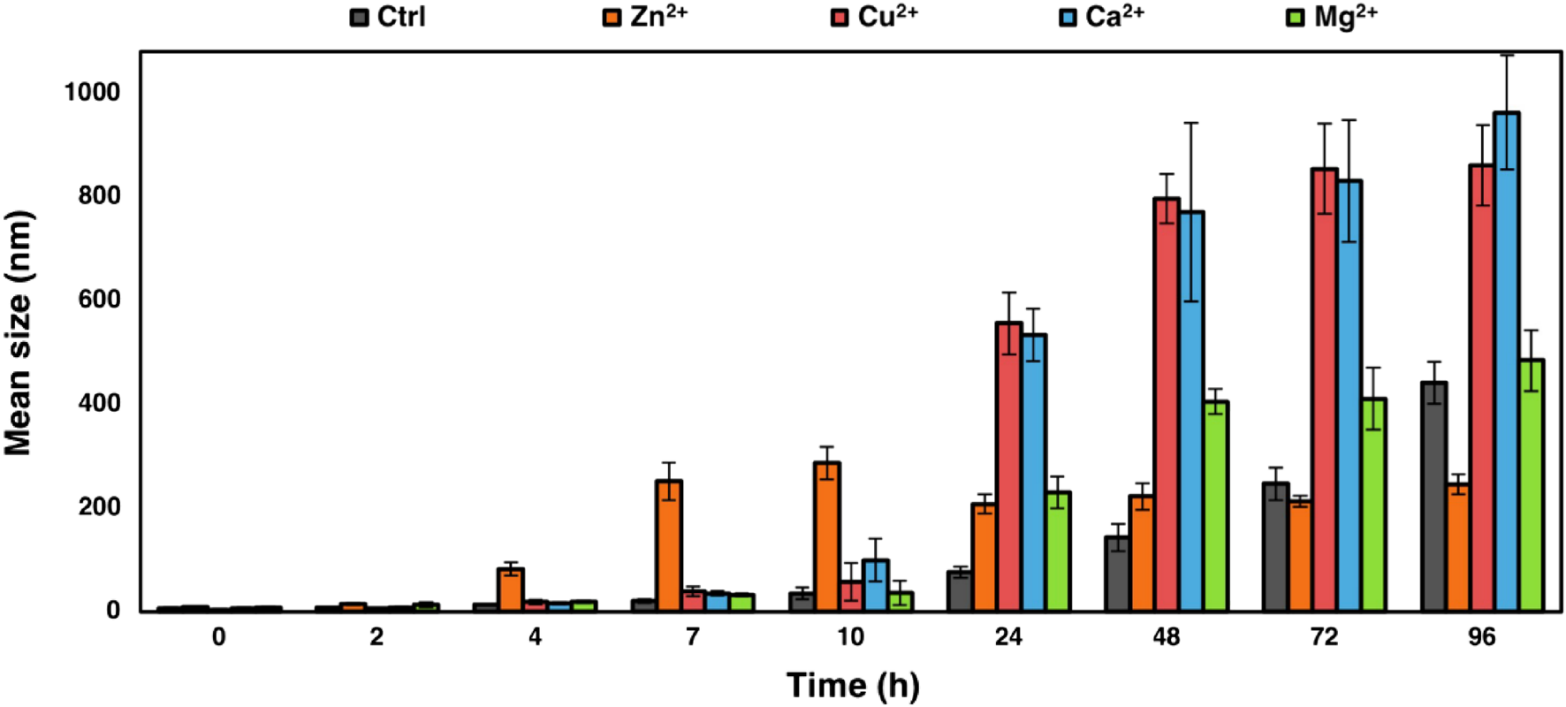
Effect of each cation on size evolution of α-Syn fibrils. The fibrils were analyzed by DLS at the denoted times. The error bars represent the standard deviations of three independent experiments.

We monitored the size evolution of the fibrils using DLS in the comparable conditions with kinetic studies. Figure 4 displays the mean size (nm) of α-Syn fibrils formed through a time scale. α-Syn aggregates formed in presence of Zn^2+^ ions rapidly started growing after 4 h and their sizes remained nearly constant after 7 h. However, Cu^2+^, Ca^2+^, and Mg^2+^ presented delayed increase in size along with longer fibrils compared to Zn^2+^. Zn^2+^ led to formation of the shortest fibrils while, Cu^2+^ and Ca^2+^ resulted in formation of the longest fibrils.

**Figure 5:**
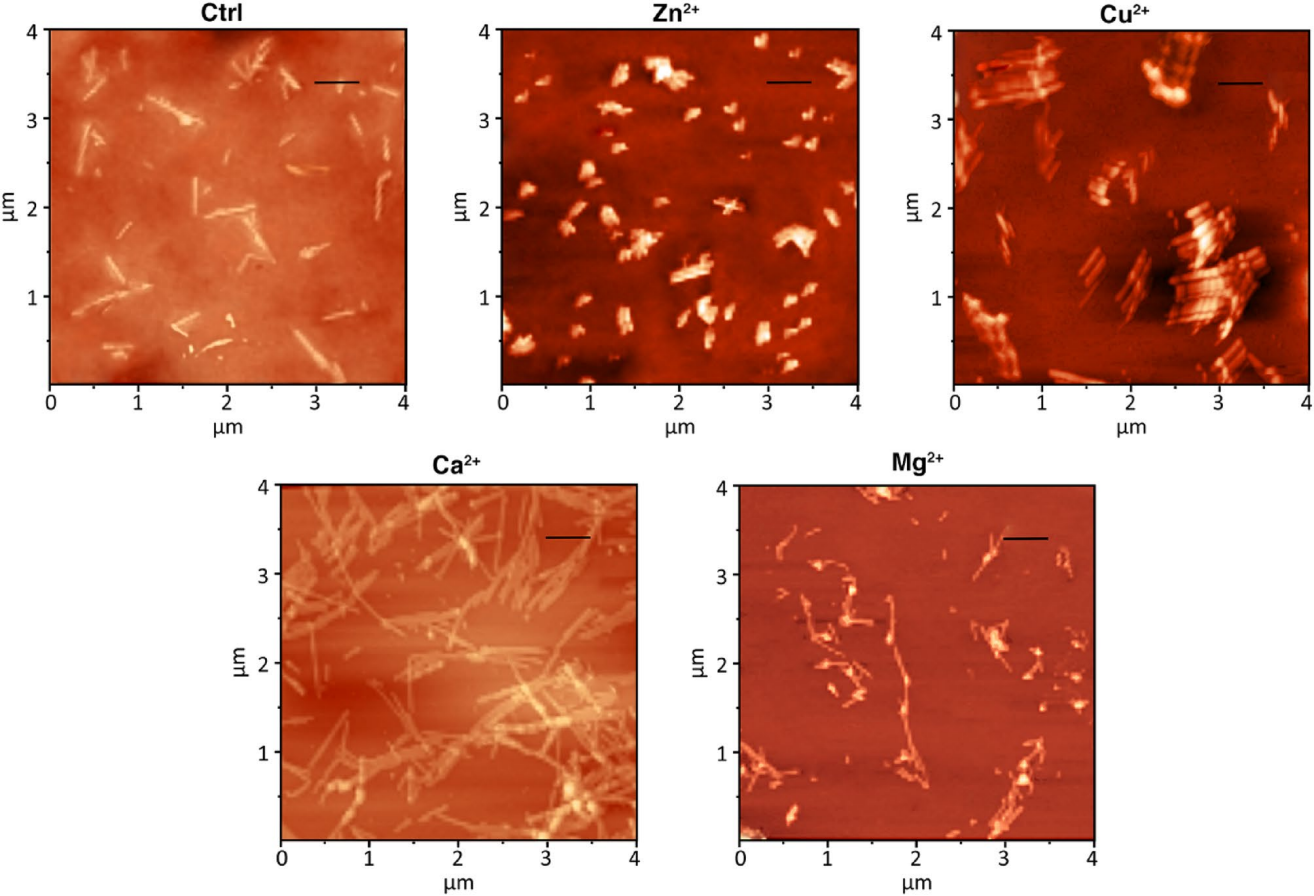
AFM images of α-Syn fibrils formed in the presence and absence of each studied cation. The end stage fibrils formed in Tris buffer or in the presence of Zn^2+^, Cu^2+^, Ca^2+^, and Mg^2+^ were analyzed. The scale bars represent 500 nm.

Since a remarkable difference in the lengths of α-Syn fibrils formed in the presence and absence of Zn^2+^, Cu^2+^, Ca^2+^, and Mg^2+^ was detected, further analysis on the size and morphology of the fibrils was performed using atomic force microscopy. AFM images were obtained from final stage fibril samples (Fig. 5). Fibril lengths were quantified using Nova software and displayed in Table 2. Interestingly, it was observed that except for the Zn^2+^, the other samples presented well-defined mature fibrils with various distribution of lengths and arrangements. Presence of Zn^2+^, Cu^2+^, and Ca^2+^ resulted in significant differences in the length of fibrils compared to the control (*p* < 0.05). Mg^2+^ did not cause a significant difference compared to the control condition (*p* = 0.81). Presence of Zn^2+^ resulted in a striking difference in length of the fibrils compared to the control and the other three metal ions (*p* < 0.05). It seemed that Zn^2+^ caused formation of shorter fibrils. In contrast, Ca^2+^ induced formation of longer fibrils compared to the control and other fibrils (*p* < 0.05).

**Table 2:** Average lengths of α-Syn fibrils formed in presence and absence of cations. The lengths were determined via analyzing AFM images using Nova software (version 1.26.0.1443). * *p* < 0.05 significant difference compared to the control group. **p < 0.05 significant difference compared to the Zn^2+^ group. ***p < 0.05 significant difference compared to the Cu^2+^ group. ****p < 0.05 significant difference compared to the Ca^2+^ group.

	Control	Zn^2+^	Cu^2+^	Ca^2+^	Mg^2+^
Average length (µm)	0.48 ± 0.09	0.20 ± 0.04	0.72 ± 0.31	0.94 ± 0.21	0.49 ± 0.22
Statistical significance vs. control		*	*	*	n.s
Statistical significance vs. Zn^2+^			**	**	**
Statistical significance vs. Cu^2+^				***	***
Statistical significance vs. Ca^2+^					****

### Zn^2+^ ions induced formation of nontoxic α-synuclein fibrils

**Figure 6:**
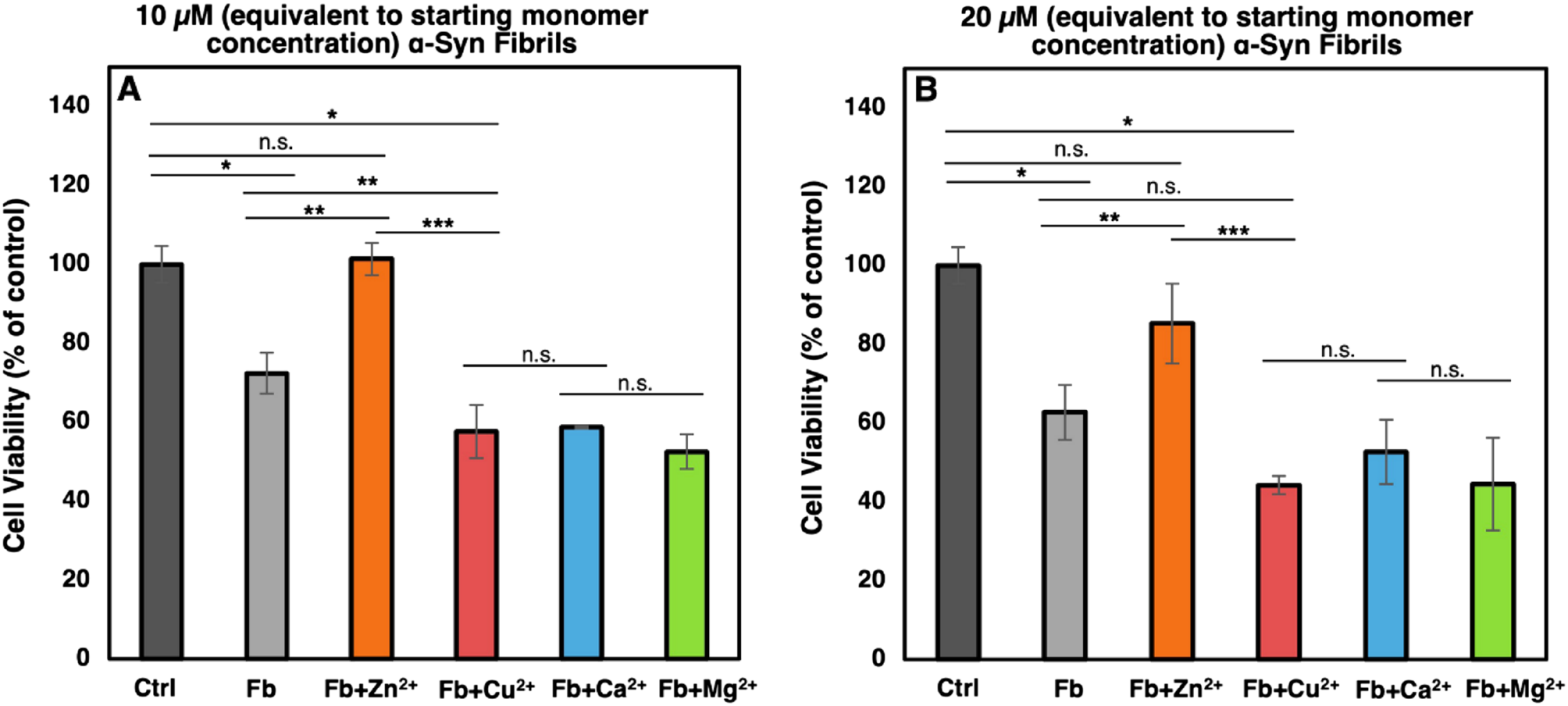
Cytotoxicity of fibrils formed in the presence and absence of each metal ion. SH-SY5Y cells were treated with α-Syn fibrils formed in Tris or in presence of the metal ions. The end stage fibrils were separated by centrifugation and after correction for concentration were used in cytotoxicity studies. The cells were treated by (**A**) 10 µM and (**B**) 20 µM of fibrils (corresponding to the starting monomer concentration). Cell viability was determined based on ability of cells to metabolize MTT. Fb: α-Syn fibrils. * *p* < 0.05 significant difference compared to the control group. **p < 0.05 significant difference compared to the Fb group. ***p < 0.05 significant difference compared to the Fb + Zn^2+^ group. “n.s.” denotes “not significant” difference.

Significant differences between the fibrils formed in the presence and absence of Zn^2+^, Cu^2+^, Ca^2+^, and Mg^2+^ stimulated us to check the cytotoxicity of fibrils using MTT assay (Fig. 6). Fibrils formed in tris buffer or in the presence of Cu^2+^, Ca^2+^, and Mg^2+^ ions resulted in a significant decrease in cell viability at 10 and 20 µM concentration, what remains in agreement with earlier published reports^26–28^. In a complete contrast, fibrils formed in the presence of Zn^2+^ ions did not provide a significant change in the viability of the cells at both concentrations. Although, we detected a significant difference between viability of the cells treated with the fibrils formed in the presence of Zn^2+^ ions compared to the fibrils formed in the presence of the other three cations, we observed no significant difference between viability of the cells treated with the fibrils formed in the presence of Cu^2+^, Ca^2+^, and Mg^2+^ ions at both concentrations. We also checked the cytotoxicity of the studied cations at the concentration the cells receive when treated with α-Syn fibrils and detected no toxicity (data not shown).

## Discussion

There has been a considerable attention on the effect of metal ions on α-Syn fibril formation^10,15,29,30^. In the present study, it was observed that different cations can trigger the formation of fibrils with different kinetics and morphology, which leads to various cytotoxicities.

Studies of fibrillation kinetics demonstrated that the cations induced acceleration of fibril formation with shorter lag phase along with higher *k*_*app*_, what suggests shorter nucleation times and higher rates of elongation (Fig. 1A and Table 1). The Zn^2+^ was the most effective in accelerating fibril formation compared with other studied cations. The results of this study are consistent with the pioneering work published by Uversky et al., which indicated stimulation of α-Syn fibril formation and also induction of conformational changes in α-Syn by cations^10^. During years of intense research on the interactions of various metal ions with α-Syn, it has been observed that Zn^2+^ binds mainly to the C-terminal region. His50 in the N terminal unstructured region has also been determined as a binding site for Zn^2+^ ions^16^. Exploring the interaction between α-Syn and Cu^2+^ has revealed 1MDVFMKGLS9 and 48VAHGV52 as the main binding sites of Cu^2+^ in the N-terminal domain of α-Syn^6,15^. Studies of the interactions between Ca^2+^ and Mg^2+^ with α-Syn indicated the acidic C-terminal region as the binding site^17^. Therefore, the differences in the binding mode and the engaged amino acids can explain the differences observed in fibrillation kinetic parameters in the presence and absence of various cations. The higher aggregation propensity of α-Syn upon metal ions binding is due to the reduction of electrostatic charges in the negatively charged C-terminal region, what leads to different degrees of neutralization of the charged N- and C-terminal regions and consequently the more energetically favorable fibril formation^15,31,32^. Therefore, the observed differences in the kinetic parameters of fibrils formed in the presence of various cations can be attributed to the variations in the binding sites and unique nature of each cations interaction with α-Syn monomers.

We also observed formation of more hydrophobic clusters and β-sheets through ANS and CR binding studies, respectively, in fibrils produced in presence of the tested cations (Fig. 1B–D), what led us to the conclusion that the assemblies formed in the presence of various cations were of amyloid nature. According to CR binding data, there was no considerable difference between the amount of fibrils formed in the presence of various cations. This along with the observed different lag times and rates of α-Syn fibrillation in the presence of Zn^2+^, Cu^2+^, Ca^2+^, and Mg^2+^ ions implied the formation of different types of fibrils.

Accumulation of β-sheets and amyloid nature of the assemblies formed in the presence and absence of various cations were analyzed using CD spectroscopy (Fig. 2). The change from disordered structure in the α-Syn monomer to the dominance of β-sheet-rich structures in the fibrils formed in the presence or absence of various cations was implied through appearance of the expected minimum at around 218 nm ^21^. In order to validate the data obtained by CD on the secondary structure of the polymorphically different fibrils and in order to confirm the amyloid nature of the fibrils, we proceed to ATR-FTIR spectroscopy (Fig. 3). The amide I band (1700–1600 cm^−1^) is mainly attributed to the C = O stretching vibration due to hydrogen bonding and distortion of amide linkages^23^. Our ATR-FTIR results were in good accordance with CD results, what showed accumulation of β-sheet structures.

We studied the size evolution of the fibrils using DLS (Fig. 4) and found a parallel trend with the kinetic studies. α-Syn aggregates formed in the presence of Zn^2+^ ions showed a fast grow, while their sizes remained nearly constant after a short time indicating the exhaustion of α-Syn monomers. However, Cu^2+^, Ca^2+^, and Mg^2+^ presented delayed increase in their sizes and at the endpoint they resulted to obviously longer fibrils in comparison with Zn^2+^. These results were in complete correspondence with our kinetic results and confirmed this conclusion that Zn^2+^ cause more nuclei formation which leads to shorter nucleation time and faster elongation.

Facing this variation in the sizes of the fibrils, we proceeded to AFM studies to check their morphology. The AFM images (Fig. 5) and the obtained fibril lengths (Table 2) were in good agreement with DLS data in that the fibrils formed in the presence of Zn^2+^ were the shortest, whereas the fibrils formed in the presence of Ca^2+^ were the longest. All the assemblies were needle like, which implied their amyloid nature.

Therefore, we had polymorphically different fibrils and thought that morphologically different fibrils could display different levels of cytotoxicity. According to previous studies, α-Syn fibrils released from dead neurons into extracellular space can be cytotoxic to neighboring neurons^33^. We observed cytotoxicity of α-Syn fibrils formed in the absence of metal ions, which was comparable to the published reports^26,27^. We also observed higher cytotoxicity of α-Syn fibrils formed in the presence of Cu^2+^, Ca^2+^ and Mg^2+^. Enhanced cytotoxicity of α-Syn fibrils formed in the presence of Cu^2+^ has also been reported previously^28^. However, nontoxicity of the short fibrils induced by Zn^2+^ was interesting. One may see this was in contrast with the reported observations that shorter fibrils are more cytotoxic compared to longer fibrils due to higher release of toxic oligomers (type B, as referred by Cascella et al.)^34^. Cascella et al. used fibrils in their study that were produced in the absence of any effective ligand, which may interfere with the structure of the formed oligomers, and release of toxic oligomers from their ends. They cleaved the fibrils through sonication to achieve shorter fibrils with more fibril ends leading to higher release of toxic oligomers. Whereas this possibility can be considered that the fibrils formed in the presence of Zn^2+^ could be more stable and release the oligomers with a lower rate or even delivering the non-toxic oligomers. On the other hand, there are a number of receptors at the cell membrane, which bind to β-sheet rich conformers and mediate their neurotoxic effect such as the cellular prion protein^35^. These receptors may have less affinity to the α-Syn conformers formed in the presence of Zn^2+^ compared to the other types of fibrils used in the current study. Alternatively, polymorphically different fibrils could have different seeding activities once entered the cells^36^. According to our unpublished data, the polymorphically different fibrils formed in the presence of various cations can imprint their morphology in the newly formed fibrils. However, studying the mechanism of non-toxicity of the fibrils formed in the presence of Zn^2+^ ions is beyond the scope of this paper and needs to be addressed in further studies.

**Figure 7:**
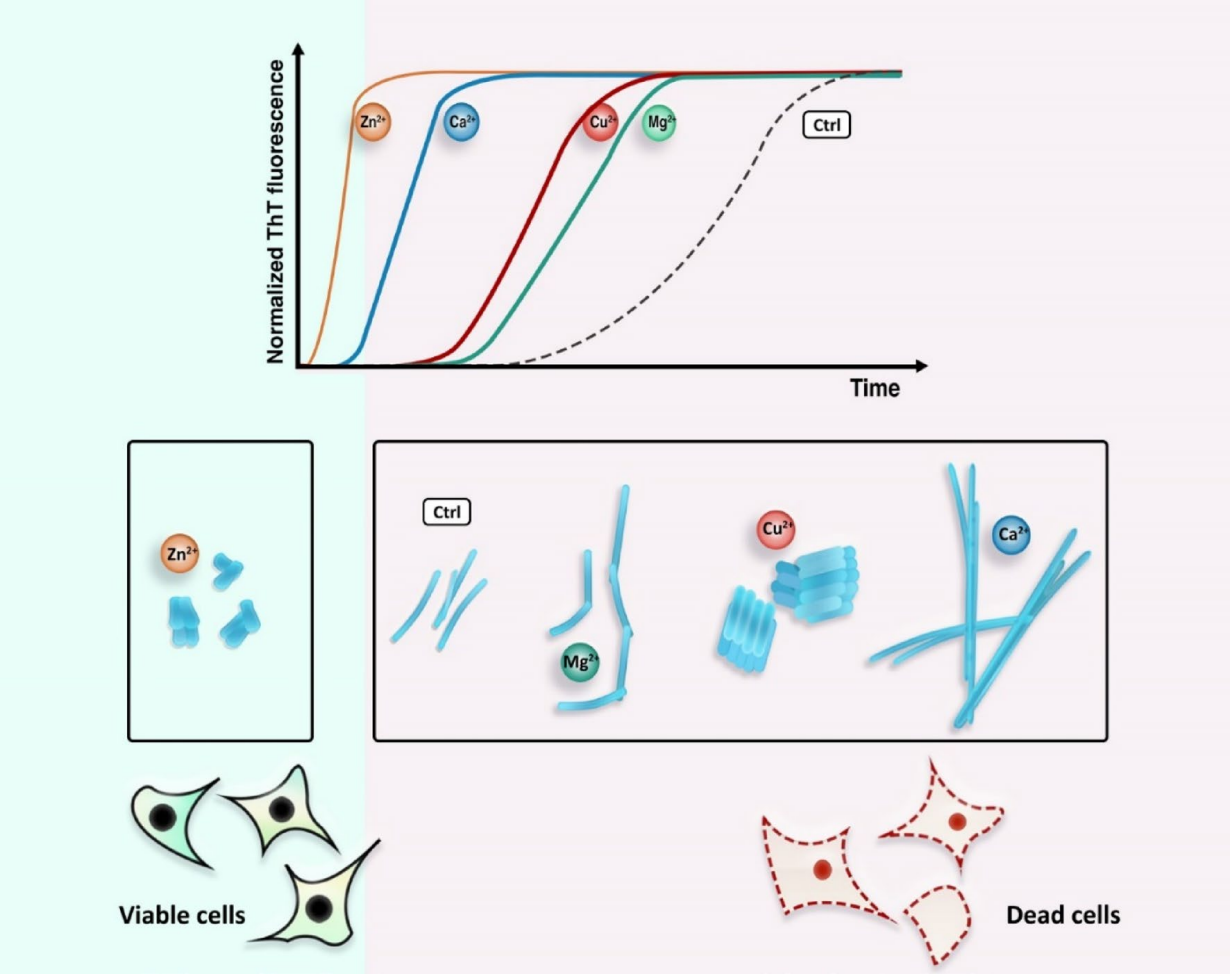
Schematic representation displaying the effect of various cations on α-Synuclein fibril formation. Various metal ions induced accelerated rate of α-Syn fibrillation and formation of polymorphically different fibrils. The fibrils produced in the presence of Zn^2+^ ions were less cytotoxic compared to the fibrils produced in the absence of cations or formed in the presence of Cu^2+^, Ca^2+^, and Mg^2+^. The schematic image was drawn using Photoshop CS5 software.

Figure 7 displays a schematic representation of the findings in the current paper. To conclude, fibrils produced in the presence of Zn^2+^, Cu^2+^, Ca^2+^, and Mg^2+^ions presented different kinetic parameters, morphology, and cytotoxicity. These can be tracked down to the different mode and binding sites of each cation’s interaction with α-Syn monomers. Particularly, in the case of Zn^2+^ ions we observed no cytotoxicity of the corresponding fibrils although that the fibrils were shorter. Therefore, the notion that the shorter fibrils are more cytotoxic than the longer fibrils cannot always be true. However, this article presented a number of hypotheses in order to explain this observation, further studies are needed to unravel the mechanism behind the non-toxicity of the shorter fibrils produced in the presence of Zn^2+^ ions.

## Materials and methods

### Materials

Thioflavin T (ThT), tris(hydroxymethyl)aminomethane, phenylmethylsulfonyl fluoride (PMSF), Congo red, 8-Anilino-1-naphthalenesulfonic acid (ANS), and 1-(4,5-Dimethylthiazol-2-yl)-3,5-diphenylformazan (MTT) were purchased from Sigma (St.Louis, MO, USA). KCl, NaCl, ZnCl_2_, CuCl_2_, CaCl_2_, MgCl_2_, ethylenediaminetetraacetic acid (EDTA), ammonium sulfate, NaH_2_PO_4_, Na_2_HPO_4_, and KH_2_PO_4_ were obtained from Merck. pT7-7 asyn WT plasmid containing α-Syn gene was obtained from addgene. Isopropyl ß-D-1-thiogalactopyranoside (IPTG) was purchased from CinnaGen. The HiTrap Q FF anion exchange chromatography column was from GE Healthcare. Dulbecco's Modified Eagles Medium (DMEM), fetal bovine serum (FBS), and trypsin were all from Gibco. Penicillin–streptomycin solution was purchased from BIO-IDEA. The cell culture flasks and plates were obtained from SPL.

### Expression and purification of α-synuclein

α-Syn was expressed in *Escherichia coli* BL21(DE3) transfected with pT7-7 asyn WT plasmid according to Hoyer et al*.* with some modifications^37,38^. Briefly, pT7-7 asyn WT plasmid containing *E. coli* cells were grown in Luria broth (LB) overnight in the presence of 100 µg/ml ampicillin and used as a pre-culture next day. Upon reaching the OD_600_ of 0.6, the cells were induced to express α-Syn using 1 mM IPTG for 4 h, while the culture flasks were incubated in 37 °C and shaken at 180 rpm. Then, the cells were harvested by centrifugation at 6000 rpm for 5 min at 4 °C to obtain the cell pellet. Following re-suspension of the cell pellet in lysis buffer (20 mM Tris base pH 8.0, 1 mM EDTA, and 1 mM PMSF), sonication at 50 W (10 s on and 10 s off) was performed. Then, the cells suspended in the lysis buffer were placed in boiling water for 20 min followed by centrifugation at 18,000 × g for 30 min at 4 °C. The supernatant was collected and ammonium sulfate was slowly added until reaching 0.36 g/ml. Following stirring for 30 min at 4 °C, the suspension was centrifuged at 18,000 × g for 20 min at 4 °C. The pellet was resuspended in 20 mM Tris buffer (pH 8.0) and loaded onto a HiTrap Q FF anion exchange chromatography column. α-Syn was eluted at 300 mM NaCl and its purity was confirmed by SDS-PAGE. The purified α-Syn was dialyzed against 20 mM Tris base buffer (pH 7.5) and its concentration was determined by measuring its absorbance at 275 nm (ε_275_ = 5600 M^-1^ cm^−1^)^7^. It is worth mentioning that the purified α-Syn used in our studies does not contain any additional motifs, *i.e.* His tag, that could interact with the metal ions used in our experiments.

### Fibril formation

To study the effect of different cations (Zn^2+^, Cu^2+^, Ca^2+^, and Mg^2+^) on α-Syn fibril formation, 100 µM of the protein in 20 mM Tris buffer (pH 7.5) was incubated in the presence and absence of 500 µM of each cation at 37 °C under 1000 rpm stirring. As a control α-Syn was incubated in Tris buffer alone. Kinetic studies were performed followed by characterization of the fibrils upon completion of the fibril formation.

### Thioflavin T fluorescence assay

Kinetic of α-Syn fibril formation was assessed in the presence of 0 and 500 µM of each metal ion using ThT fluorescence assay^39,40^. ThT fluorescence assays were carried on a Cary Eclipse VARIAN fluorescence spectrophotometer (Mulgrave, Australia). The measurements were performed by adding 10 µl of 100 µM α-Syn to 490 µl of 25 µM ThT solution followed by 5 min incubation in room temperature. ThT sample was prepared as a 10 mM stock solution, filtered through a 0.2 µm filter, and diluted to 25 µM in 25 mM sodium phosphate buffer (pH 6.5). ThT was excited at 450 nm and the emission was monitored at 465–550 nm. The excitation and emission slits were set at 5 and 10 nm, respectively. The obtained data from ThT fluorescence were fitted using the following equation ^41^:

1 $$ F = F_{\min } \left( {\frac{Fmax}{{1 + e^{{ - \left[ {\frac{{\left( {t - t0} \right)}}{\tau }} \right]}} }}} \right) $$

where F_min_ and F_max_ define the fluorescence intensities at time 0 and saturation phase of incubation, respectively. t is the incubation time and t_0_ is the time that 50% of maximal fluorescence is obtained. The value of τ was determined by nonlinear regression. Apparent growth rate constant (*k*_app_) and lag phase time (t_lag_) were calculated as 1/τ and t_0_ − 2τ, respectively^42^. The kinetic studies were performed in triplicate.

### ANS binding assay

For each measurement, 490 µl ANS solution was mixed with 10 µl of end stage α-Syn fibrils. ANS was excited at 350 nm and emission spectra were recorded between 400 and 600 nm. Excitation and emission slits were set at 5 and 10 nm, respectively.

### Congo red adsorption

Congo red solution was prepared in Congo red buffer containing 5 mM potassium phosphate and 150 mM NaCl (pH 7.5). Upon completion of α-Syn fibril formation, 25 µl of each sample was added to 475 µl of 20 µM Congo red solution. Following 30 min incubation in room temperature, absorbance spectra in the range 400–600 nm were collected. The amount of Congo red bound to fibrils, as an index of amount of fibrils, was determined using the following equation ^43^:

2 $$ {\text{CRB }}\left( {\text{M}} \right) \, = \, \left( {{\text{A54}}0/{25295}} \right)/\left( {{\text{A488}}/{463}0{6}} \right) $$

where CRB defined the molar concentration of bound Congo red, and 25,295 and 46,306 present the molar extinction coefficients of bound and unbound Congo red, respectively.

### Circular dichroism spectroscopy

Secondary structural changes of α-Syn at various time points during fibril formation in the presence of 0 and 500 µM of each metal ion (Zn^2+^, Cu^2+^, Ca^2+^, and Mg^2+^) was studied using far UV (190–260 nm) circular dichroism spectroscopy on an AVIV 215 spectropolarimeter. The protein was diluted to a final concentration of 20 µM and placed in 0.1 cm path length cuvettes. The CD data were finally analyzed using CDSD software.

### Attenuated total reflection-Fourier transform infrared spectroscopy

ATR-FTIR measurements were taken using an AVATAR Thermo FTIR at room temperature at a resolution of 1 cm^−1^. α-Syn fibrils samples were centrifuged for 40 min at 20,000 × g and 4 °C and concentrated before ATR-FTIR measurements. The spectra of water were subtracted from the raw data. The obtained data were baseline-corrected, smoothed, and normalized using GraphPad Prism 8. The amide I band (1700–1600 cm^−1^) of the spectra were fitted based on a Gaussian model using OriginPro 2022.

### Dynamic light scattering

To analyze the size distribution of α-Syn assemblies at various time points during fibril formation, we performed dynamic light scattering (DLS) measurements using a Zeta plus (Brookhaven Instruments). The solutions were filtered through a 0.2 µm syringe filter. Final concentration of each sample was 8 µM (starting concentration equivalent) in the cuvette. The measurements were performed by a laser of 657 nm and at fixed detector angle of 90° in room temperature (25 °C). DLS experiments were performed in triplicate.

### Atomic force microscopy

α-Syn fibrils formed in the presence and absence of 500 µM of each studied cation were also analyzed by atomic force microscopy. Briefly, 10 µl of 100 fold diluted α-Syn fibril samples were placed on a freshly cleaved mica and dried at room temperature. Then, the images were obtained in semicontact mode using an Atomic Force Microscopy (NTEGRA, NT-MDT, Russia) followed by processing the images by Nova software (version 1.26.0.1443). For each sample 23–27 measurements were performed.

### Cell studies

Human neuroblastoma SH-SY5Y cells were a kind gift from Dr. Saeed Karima (Department of Clinical Biochemistry, Shahid Behehshti University of Medical Sciences, Tehran, Iran). The cells were cultured in high glucose DMEM supplemented with 10% fetal bovine serum and 1% penicillin and streptomycin. SH-SY5Y cells were cultured in T25 flasks (SPL, South Korea) at a density of 20,000 cell/cm^2^ and subcultured using 0.25% (w/v) trypsin and 0.03% (w/v) EDTA in Dulbecco's Phosphate Buffered Saline (DPBS) to detach cells. The cells were incubated at 37 °C in a humidified atmosphere (95%) along with 5% CO_2_.

Effect of α-Syn fibrils formed in the presence or absence of 500 µM Zn^2+^, Cu^2+^, Ca^2+^, and Mg^2+^ on SH-SY5Y neuroblastoma cells were evaluated using MTT assay. The cells were harvested and seeded at 5,000 cells/well in a 96-well cell culture plate (SPL, South Korea) and incubated for 24 h at 37 °C to allow cells to attach. Then, the cells were treated with 0, 10, and 20 µM of fibrils formed in the presence or absence of 500 µM Zn^2+^, Cu^2+^, Ca^2+^, and Mg^2+^ and incubated for 24 h. Then, the culture media were aspirated and the cells were treated with fresh media containing 0.25 mg/ml MTT. Following incubation for 3 h at 37 °C, the culture media were removed and 100 µl DMSO was added to each well to dissolve the formed formazan crystals. Absorbance of the formed colorful solution was measured at 570 nm using an Elisa reader spectrophotometer (BioTek, USA). Cell viability was calculated using the following equation ^44^:

3 $${\text{Viable cells (\% ) = }}\left( {{\text{Abs of sample}} \times {100}} \right){/}\left( {\text{Abs of control}} \right)$$

### Statistical analysis

All the results were average of at least three independent experiments and represented as mean ± standard error of mean (SEM). The statistical analysis was carried out using Microsoft Office Excel Version 2016. One-way analysis of variance (ANOVA) and Tukey’s post-hoc analysis were used to compare means for continuous variables. A *p*-value of 0.05 was considered as the level of statistical significance.

## Data Availability

The data that support the findings of this study are available from the corresponding author upon reasonable request.
